# Lower Education Level Is a Risk Factor for Peritonitis and Technique Failure but Not a Risk for Overall Mortality in Peritoneal Dialysis under Comprehensive Training System

**DOI:** 10.1371/journal.pone.0169063

**Published:** 2017-01-05

**Authors:** Hyo Jin Kim, Joongyub Lee, Miseon Park, Yuri Kim, Hajeong Lee, Dong Ki Kim, Kwon Wook Joo, Yon Su Kim, Eun Jin Cho, Curie Ahn, Kook-Hwan Oh

**Affiliations:** 1 Department of Internal Medicine, Dongguk University Gyeongju Hospital, Gyeongju-si, Gyeongsangbuk-do, Korea; 2 Medical Research Collaborating Center, Seoul National University Hospital, Seoul, Korea; 3 Department of Internal Medicine, Seoul National University Hospital, Seoul, Korea; 4 Department of Internal Medicine, Hongseong Medical Center, Hongseong, Korea; Kaohsiung Medical University Hospital, TAIWAN

## Abstract

**Background:**

Lower education level could be a risk factor for higher peritoneal dialysis (PD)-associated peritonitis, potentially resulting in technique failure. This study evaluated the influence of lower education level on the development of peritonitis, technique failure, and overall mortality.

**Methods:**

Patients over 18 years of age who started PD at Seoul National University Hospital between 2000 and 2012 with information on the academic background were enrolled. Patients were divided into three groups: middle school or lower (academic year≤9, n = 102), high school (9<academic year≤12, n = 229), and higher than high school (academic year>12, n = 324). Outcomes were analyzed using Cox proportional hazards models and competing risk regression.

**Results:**

A total of 655 incident PD patients (60.9% male, age 48.4±14.1 years) were analyzed. During follow-up for 41 (interquartile range, 20–65) months, 255 patients (38.9%) experienced more than one episode of peritonitis, 138 patients (21.1%) underwent technique failure, and 78 patients (11.9%) died. After adjustment, middle school or lower education group was an independent risk factor for peritonitis (adjusted hazard ratio [HR], 1.61; 95% confidence interval [CI], 1.10–2.36; *P* = 0.015) and technique failure (adjusted HR, 1.87; 95% CI, 1.10–3.18; *P* = 0.038), compared with higher than high school education group. However, lower education was not associated with increased mortality either by as-treated (adjusted HR, 1.11; 95% CI, 0.53–2.33; *P* = 0.788) or intent-to-treat analysis (*P* = 0.726).

**Conclusions:**

Although lower education was a significant risk factor for peritonitis and technique failure, it was not associated with increased mortality in PD patients. Comprehensive training and multidisciplinary education may overcome the lower education level in PD.

## Introduction

Hemodialysis (HD) and peritoneal dialysis (PD) are two options of dialysis modality for renal replacement therapy (RRT). As a home-based treatment, PD requires patients’ comprehensive understanding and management abilities for PD procedure [1, 2]. These factors may relate to socioeconomic status and education levels. Several social factors including education level above elementary school and flexibility in work are significantly associated with choosing PD as the first line dialysis modality, compared to HD [3].

PD-related peritonitis is one of the major complications of PD and it can influence technique failure, morbidity and mortality in PD patients [4, 5]. There were several previous reports on the association between education level and peritonitis, technique failure, and mortality in PD patients. Lower education level below elementary school [6] or patients from lower education area [7] were a risk factor for peritonitis. Risk of technique failure and mortality with respect to the education levels varied among several reports. Education level was associated [8] or not associated [7] with PD technique survival. In addition, education level was associated [9] or not associated [8] with patient death in previous studies. Yang *et al*. [10] emphasized that family members’ education level was important for mortality outcome of PD patients. There were conflicting results as regards the association between education level and PD outcomes.

As a home-based therapy, PD depends more heavily on the patients’ role than HD. Lower education status could be a more crucial issue for PD. In our previous report, we have shown that multidisciplinary pre-dialysis education (MPE) for subjects with chronic kidney disease improved patient outcomes in terms of urgent dialysis, cardiovascular events, and infection [11]. Therefore, in the present study, we analyzed the association between lower education level and various patient outcomes such as patient survival, technique survival, and peritonitis under a structured patient education system for PD patients.

## Materials and Methods

### Study subjects

**Fig 1:**
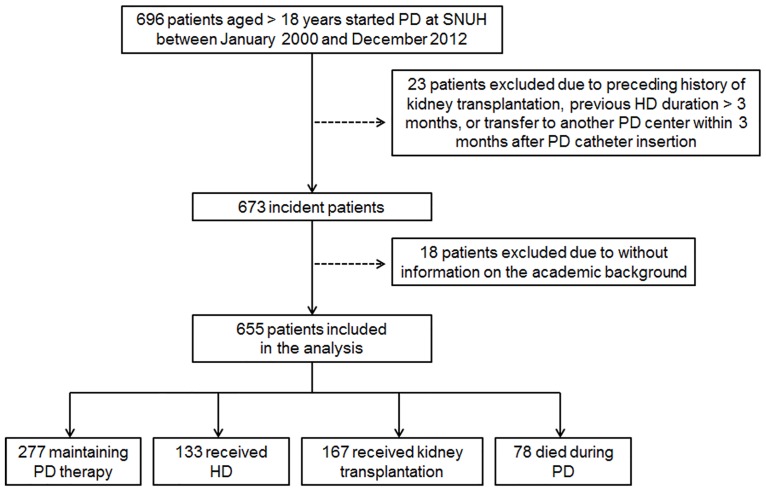
Flow chart of study subject selection and final modality and status of patients in the study. PD, peritoneal dialysis; SNUH, Seoul National University Hospital; HD, hemodialysis.

The present study is a single-center retrospective analysis of prospectively collected registry data. Individualized multidisciplinary education program is provided to patients prior to initiating PD in Seoul National University Hospital (SNUH) [11]. As a regular medical practice, the patient and family members are informed by one-on-one lecture focused on the benefits or complications, and or outcomes of RRT, options for RRT, medication education, and dietary education. In addition, nephrologists evaluate whether the patients’ status–visual disturbance, presence of dementia, housing sanitation, family support, and so on- is suitable for performing PD. Only after such evaluation, elderly patients who are suitable for PD are started on PD. Patients with severe visual disturbance or dementia who could not perform PD procedure by themselves were not considered to initiate PD. After initiating PD, patients take individualized education sessions focused on PD-related procedure such as exchanging dialysate bag and exit site care by aseptic sterilization and antibiotics ointment by professionally trained nurses. Data for all the subjects who initiate PD therapy in the SNUH are managed prospectively by the SNUH PD Registry. Data captured prospectively by SNUH PD Registry include age, sex, socioeconomic status, etiology of renal failure, history of previous RRT, comorbidity, biochemistry, peritoneal function test, PD adequacy, peritonitis, technique failure, mortality and so on. Six hundred ninety six patients over 18years of age started PD at SNUH between January, 2000 and December, 2012. After initiating PD, each patient agreed to the use of his/her demographic and laboratory data for future studies. Among them, 23 patients were excluded because of preceding history of kidney transplantation, HD duration >3 months before starting PD, or transfer to another PD center within three months after PD catheter insertion. Finally, 655patients with information on the academic background were analyzed (Fig 1). This study was approved by the Seoul National University Hospital Institutional Review Board and the need for informed consent from the patients was waived because of the retrospective design of the study. Patient information was anonymized and de-identified prior to analysis. All clinical investigations were conducted in accordance with the guidelines of the 2008 Declaration of Helsinki.

### Clinical data collection

The baseline demographic data such as age, gender, academic background, PD staring year, healthcare insurance status, comorbidities, cause of end stage renal disease (ESRD), and body mass index (BMI) and biochemical data immediately before PD catheter insertion were investigated. PD starting year was divided into two categories—2000 to 2006 and 2007 to 2012. The Korean national healthcare system is divided into Medical insurance and Medicaid covered for the poor. Comorbidities of patients are evaluated by Davies comorbidity score [12, 13]including ischemic heart disease, peripheral vascular disease, left ventricular dysfunction, malignancy, diabetes mellitus (DM), systemic collagen vascular disease, and others. We assessed DM as a single variable and calculated comorbidity score guided by Davies comorbidity index excluding DM. Comorbidity score was categorized as three risk groups-low (score 0), intermediate (score 1–2), and high (score ≥ 3). Serum levels of hemoglobin, C-reactive protein (CRP), and albumin were measured by using routine laboratory methods. Creatinine was measured by isotope dilution mass spectrometry (IDMS) reference method [14]. Estimated glomerular filtration rate (eGFR) at the dialysis initiation was calculated by abbreviated 4-variable Modification of Diet in Renal Disease (MDRD) study equation [15], using IDMS-traceable serum creatinine assay performed immediately before starting dialysis.

### Outcome measurement

Based on the academic years, patients were categorized into 3 groups; middle school or lower (academic year ≤9 years), high school (9 years < academic year ≤ 12 years), and higher than high school (academic year > 12 years). Subjects were followed until the time of death, loss to follow-up, or April 1^st^, 2014, whichever came first. Primary outcome was all-cause mortality analyzed using as-treated (AT) approach with respect to the academic groups. For AT analysis, patients were censored at the time of follow-up loss or 90 days after they switched to HD or received kidney transplantation. Deaths within 90 days after modality switch were also considered as PD-related mortality. In addition to AT analysis, *post hoc* intent-to-treat (ITT) analysis was also performed in order to preclude the possibility that the subjects potentially having poorer outcomes might have been selectively censored by AT analysis, resulting in informative censoring. For ITT analysis, information on the mortality even after modality switch was included in the outcome analysis. Secondary outcomes were the first peritonitis event and technique failure with respect to the academic groups. PD related peritonitis was diagnosed by International Society for Peritonitis Dialysis guideline [16]. The incidence of peritonitis according to the causative microorganism was evaluated. PD technique failure was defined as a switch to HD from any cause including inadequate dialysis, ultrafiltration failure, intractable peritonitis such as refractory, recurrent, or fungal peritonitis, exit-site and/or tunnel infection, catheter malfunction, mechanical problems such as hernia or abdominal operation, and others such as patient’s wish. Temporary HD, less than 90 days was not included in technique failure. Patient death and kidney transplantation were regarded as competing risk events in the PD technique failure.

### Statistical analysis

Categorical variables were presented as frequencies and percentage, which were compared between education groups by Chi-square test or Fisher’s exact test. Continuous variables were evaluated with analysis of variance (ANOVA). Results are shown as mean ± standard deviation (SD) for normally distributed variables and median (interquartile range [IQR]) for variables with skewed distributions. A log transformation was used to normalize variability of the CRP. We performed Cox proportional hazards models analysis with adjustments (enter method) including variables that were significant in the univariate analysis or other clinically relevant variables to demonstrate the independent risk factors related to all-cause mortality and time-to-the first peritonitis. Higher than high school education group, which has the highest education level, was regarded as the reference group. Collinearity among variables was tested and no significant interaction was found between variables. Differences in technique failure rates were compared using a Fine and Gray model (competing risks regression) [17]. Univariate analysis using competing risk regression was performed to demonstrate the risk factors for technique failure, followed by stepwise multivariate analysis for determining significant factors based on a significant level of 0.2. After verifying the interaction of each significant variable, the final model comprised only those factors with a significance level of 0.05. SPSS Statistics software (SPSS version 18.0, Chicago, IL, USA) and R statistical software (R Foundation for Statistical Computing, Vienna, Austria) were used for statistical analysis. *P*-values <0.05 were considered statistically significant.

## Results

### Demographic and baseline clinical characteristics

A total of 655 incident PD patients were analyzed. There were 102, 229, and 324 patients in middle school or lower, high school, and higher than high school education groups, respectively. The mean age was 48.4±14.1 years and 399 (60.9%) patients were male (Table 1). Two hundred thirty three (35.6%) patients had DM. Subjects in higher than high school education group were younger (*P*< 0.001, Table 1) and there were more male patients (*P* = 0.001, Table 1). There was more patients starting PD before 2006 in middle school or lower education group *(P* = 0.002, Table 1). The level of serum albumin was higher in higher than high school education group (*P* = 0.038, Table 1). Patients supported by Medicaid service were more common in middle school or lower education group *(P* = 0.038, Table 1).

**Table 1:** The baseline clinical characteristics of patients with respect to the academic groups.

		All subjects (N = 655)	Education groups			
			Middle school or lower (≤ 9 years) (n = 102)	High school (9< ≤ 12years) (n = 229)	Higher than high school (> 12years) (n = 324)	*P-* value
Age (years)*		48.4 ± 14.1	56.5 ± 11.9^a^	48.0 ± 13.9^b^	46.2 ± 13.9^c^	< 0.001
Gender (male, n, %)		399 (60.9)	53 (52.0)	125 (54.6)	221 (68.2)	0.001
PD staring year, 2000~2006 (n, %)		318 (48.5)	66 (64.7)	106 (46.3)	146 (45.1)	0.002
Medicaid, n (%)		59 (9.0%)	14 (13.7)	26 (11.4)	19 (5.9)	0.038
BMI (g/m^2^)*		22.5 ± 3.2	23.2 ± 3.0^a^	22.4 ± 3.0^b^	22.3 ± 3.5^c^	0.05
Comorbidity (n, %)						
	DM	233 (35.6)	41 (40.2)	75 (32.8)	117(36.1)	0.409
	Hypertension	602 (91.9)	99 (97.1)	207 (90.8)	296 (91.4)	0.122
	Ischemic heart disease	94 (14.4)	19 (18.6)	29 (12.7)	46 (14.2)	0.179
	Congestive heart failure	84 (12.8)	18 (17.6)	29 (12.7)	37 (11.4)	0.152
	Peripheral vascular disease**	70 (10.7)	15 (14.7)	27 (11.8)	28 (8.6)	0.179
Comorbidity score†(n, %)						0.048
	None (0)	431 (65.8)	57 (55.9)	147 (64.2)	227 (70.1)	
	Intermediate (1–2)	210 (32.1)	41 (40.2)	79 (34.5)	90 (27.8)	
	High (≥3)	14 (2.1)	4 (3.9)	3 (1.3)	7 (2.2)	
Visual disturbance		104 (15.9)	19 (18.6)	31 (13.5)	54 (18.7)	0.436
Cause of ESRD (n, %)						0.005
	DM	214 (32.7)	41 (40.2)	70 (30.6)	103 (31.8)	
	Hypertension	125 (19.1)	29 (28.4)	43 (18.8)	53 (16.4)	
	Glomerulonephritis	189 (28.9)	12 (11.8)	72 (31.4)	105 (32.4)	
	Others	37 (5.6)	7 (6.9)	10 (4.4)	20 (6.2)	
	Unknown	90 (13.7)	13 (12.7)	34 (14.8)	43 (13.3)	
Hemoglobin (g/dL)		9.8 ± 1.5	9.9 ± 1.5	9.6 ± 1.4	9.8 ± 1.5	0.155
CRP, median (Q1, Q3) (mg/dL)		0.3 (0.1, 0.9)	0.3 (0.1, 1.1)	0.3 (0.1, 0.9)	0.3 (0.1, 0.9)	0.183
Albumin (g/dL)*		3.6 ± 0.5	3.5 ± 0.4^a^	3.5 ± 0.5^b^	3.6 ± 0.5^c^	0.038
eGFR (ml/min/1.73m^2^)		8.7 ± 3.8	9.5 ± 3.8	8.5 ± 3.6	8.6 ± 4.0	0.115

*The same letters indicate non-significant differences between groups based on Tukey B or Dunnett T3 multiple comparison test *post hoc* analysis in ANOVA

BMI, body mass index; DM, diabetes mellitus; CRP, C-reactive protein; eGFR, estimated glomerular filtration rate by MDRD equation.

**distal aortic, lower limb, and cerebrovascular disease.

^†^Davies comorbidity score including ischemic heart disease, peripheral vascular disease, left ventricular dysfunction, malignancy, systemic collagen vascular disease, other, excluding DM.

### All-cause mortality according to education level

**Fig 2:**
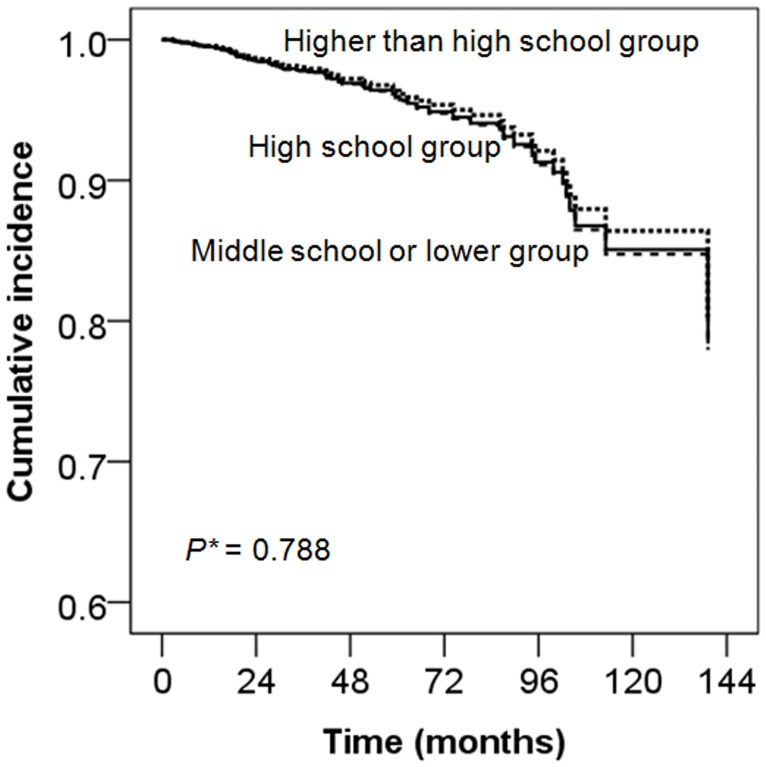
All-cause mortality according to education level. Analyzed by Cox proportional hazards models, middle school or lower education level was not associated with increased all-cause mortality, as compared to the higher than high school education (*P* = 0.788). Adjusted for age, gender, PD staring year, healthcare insurance status, DM, hypertension, comorbidity score, visual disturbance, BMI, hemoglobin, albumin, logCRP, and baseline eGFR. Middle school or lower group: academic year≤ 9 years; High school group: 9 < academic year ≤ 12 years; Higher than high school group: academic year > 12 years DM, diabetes mellitus; BMI, body mass index; CRP, C-reactive protein; eGFR, estimated glomerular filtration rate. *Middle school or lower education group compared with higher than high school education group.

For AT analysis, the median follow-up duration was 41 (IQR, 20–65) months. During follow-up period, 78 (11.9%) patients died. In the univariate analysis, all-cause mortality was not significantly different among the three education groups. However, patients in higher than high school education group were significantly younger. In a Cox proportional hazards models adjusted for age, gender, PD staring year, healthcare insurance status, DM, hypertension, comorbidity score, visual disturbance, BMI, hemoglobin, albumin, logCRP, and eGFR, middle school or lower education level was not associated with increased all-cause mortality, as compared to the higher than high school education (adjusted HR, 1.11; 95% CI, 0.53–2.33; *P* = 0.788; Table 2; Fig 2). We performed subgroup analysis categorized by age < 50 or≥ 50 years to eliminate the possible collinearity between age and many variables-such as education levels and PD starting year. There was no significant difference in all-cause mortality among the three education groups in both subgroups (*P* = 0.389 with age < 50; *P* = 0.910 with age ≥ 50; S1 File).

**Table 2:** Parameters included in the predictive equation for all-cause mortality estimated by Cox proportional hazards models.

		As-treated analysis			Intent-to-treat analysis		
Variable		Adjusted HR	95% CI	*P*- value	Adjusted HR	95% CI	*P*- value
Academic years				0.925			0.894
	**Higher than high school**	1			1		
	**Middle school or lower**	1.11	0.53–2.33	0.788	0.89	0.46–1.71	0.726
	**High school**	1.13	0.59–2.17	0.706	1.03	0.60–1.79	0.902
Gender (female *vs*. male)		1.54	0.83–2.86	0.173	1.19	0.72–1.98	0.493
Age		1.07	1.04–1.10	< 0.001	1.06	1.04–1.09	< 0.001
PD staring year (2007~2012 *vs*. 2000~2006)		0.87	0.45–1.66	0.667	0.89	0.49–1.60	0.689
Medicaid (*vs*. Medical insurance)		1.37	0.59–3.21	0.463	1.27	0.59–2.70	0.542
DM		1.87	0.94–3.73	0.074	1.88	1.07–3.32	0.029
Hypertension		2.10	0.44–10.12	0.355	2.96	0.70–12.56	0.140
Comorbidity score*				< 0.001			< 0.001
	**None (0)**	1			1		
	**Intermediate (1–2)**	5.77	2.86–11.64	< 0.001	5.31	3.02–9.33	< 0.001
	**High (≥3)**	8.40	2.56–27.48	< 0.001	7.07	2.43-20-52	< 0.001
Visual disturbance		0.91	0.45–1.85	0.792	0.94	0.50–1.75	0.840
BMI		0.95	0.86–1.04	0.265	0.97	0.89–1.05	0.419
Hemoglobin		0.96	0.76–1.23	0.767	0.99	0.82–1.20	0.926
Albumin		0.57	0.31–1.04	0.068	0.59	0.36–0.98	0.041
LogCRP		0.57	0.31–1.04	0.913	0.93	0.68–1.29	0.683
eGFR		1.05	0.98–1.13	0.158	1.01	0.95–1.08	0.683

The following variables were included to adjust by Cox proportional hazards models: age, gender, PD staring year, healthcare insurance status, DM, hypertension, comorbidity score*, visual disturbance, BMI, hemoglobin, albumin, logCRP, and eGFR. *Davies comorbidity score including ischemic heart disease, peripheral vascular disease, left ventricular dysfunction, malignancy, systemic collagen vascular disease, other, excluding DM.

PD, peritoneal dialysis; HR, Hazard ratio; CI, confidence interval; DM, diabetes mellitus; eGFR, estimated glomerular filtration rate; BMI, body mass index; CRP, C-reactive protein.

For ITT analysis, patients were followed-up for 65 (IQR, 35–100) months. There were 96(14.7%) deaths. After adjustment, there was no significant difference in all-cause mortality among the three education groups (*P* = 0.894; Table 2).

The causes of death (AT analysis) are shown in Table 3. Cardiovascular disease was the most common known cause of mortality, accounting for 33.3%. Peritonitis accounted for only 12.8% of total deaths. There was no significant difference in cause of death among the three education groups (*P* = 0.504).

**Table 3:** The causes of death with respect to education groups(As-treated analysis).

Cause, n (%)	All subjects (N = 655)	Education group			
		Middle school or lower (≤ 9 years) (n = 102)	High school (9< ≤ 12years) (n = 229)	Higher than high school (> 12years) (n = 324)	*P-* value
Peritonitis, n (%)	10 (12.8)	4 (22.2)	3 (11.1)	3 (9.1)	0.381
Other infection, n (%)	5 (6.4)	1 (5.6)	2 (7.4)	2 (6.1)	1.000
Cardiac event, n (%)	20 (25.6)	3 (16.7)	9 (33.3)	8 (24.2)	0.478
Cerebrovascular event, n (%)	6 (7.7)	1 (5.6)	0 (0)	5 (15.2)	0.081
Others or unknown, n (%)	37 (47.4)	9 (50.0)	13 (48.1)	15 (45.5)	0.958
Total	78 (100)	18 (100)	27 (100)	33 (100)	0.504

### Development of peritonitis and technique failure according to education level

**Fig 3:**
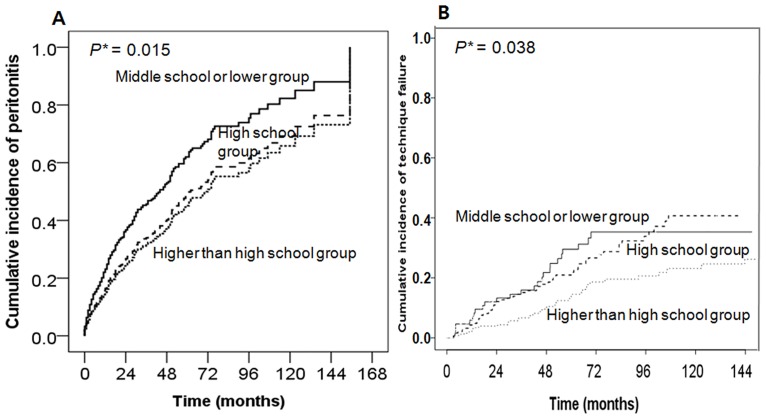
Time-to-the first peritonitis event according to education level (B) Time-to-the development of technique failure according to education level. (A) Analyzed by Cox proportional hazards models with adjustment for age, gender, PD staring year, healthcare insurance status, DM, hypertension, comorbidity score, visual disturbance, BMI, hemoglobin, albumin, logCRP, and baseline eGFR. (B) Analyzed by competing risk regression with adjustment for age and gender. Middle school or lower group: academic year ≤ 9 years; High school group: 9 < academic year ≤ 12 years; Higher than high school group: academic year > 12 years DM, diabetes mellitus; BMI, body mass index; CRP, C-reactive protein; eGFR, estimated glomerular filtration rate. *Middle school or lower education group compared with higher than high school education group.

**Fig 4:**
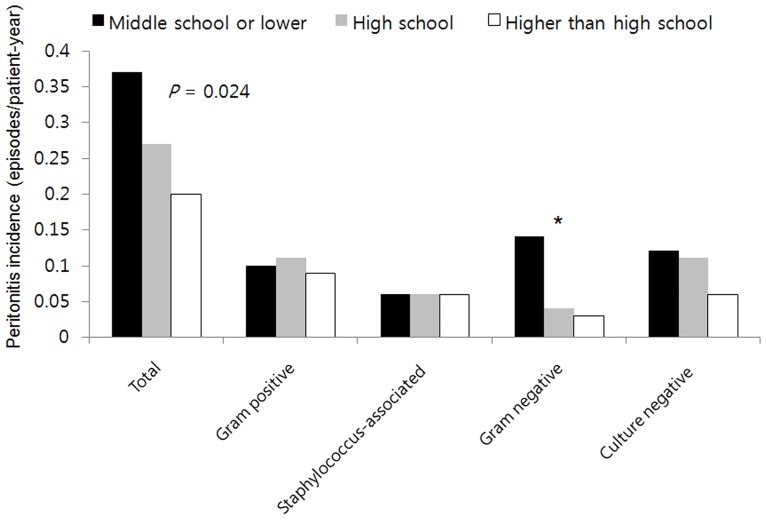
The incidences of total peritonitis episode and cause-specific peritonitis with respect to the education groups. The incidences of total (*P* = 0.024) and gram-negative peritonitis (*P* = 0.017) are higher in middle school or lower education group. However, there were no significant differences in the incidences of gram-positive (*P* = 0.718), *Staphylococcus*-associated (*P* = 0.380), and culture negative (*P* = 0.213) peritonitis. *means *P*< 0.05.

During follow-up period, 255 (38.9%) patients experienced more than one episode of peritonitis and the average time to development of first peritonitis was26.4 ± 27.2 months. A total of 475 episodes of peritonitis were observed during follow-up period. The overall incidence of peritonitis was 0.25episodes/patient-year. Fifty (49.1%), 92 (40.2%), and 113 (34.9%) patients in middle school or lower, high school, and higher than high school education groups, respectively, experienced at least one episode of peritonitis. In a Cox proportional hazards models adjusted for age, gender, PD staring year, healthcare insurance status, DM, hypertension, comorbidity score, visual disturbance, BMI, hemoglobin, albumin, log CRP, and baseline eGFR, middle school or lower education group, but not high school education group was shown to be an independent risk factor for development of peritonitis (adjusted HR, 1.61; 95% CI, 1.10–2.36; *P* = 0.015, Fig 3A) compared with the reference group. Female sex was associated with lower risk of peritonitis (adjusted HR, 0.71; 95% CI, 0.53–0.95; *P* = 0.023). In the subgroup with age ≥ 50, middle school or lower education was an independent risk factor for peritonitis (adjusted HR, 1.85; 95% CI, 1.11–3.08; *P* = 0.018, Fig B in S2 File). However, in the younger subgroup (age <50), there were no significant differences for peritonitis among the three education levels (*P* = 0.775, Fig A in S2 File). The total incidence of peritonitis and cause-specific peritonitis with respect to the education groups during study period were shown in Fig 4. The incidence of total (*P* = 0.024) and gram- negative peritonitis (*P* = 0.017) is higher in middle school or lower education group. However, there were no significant differences in the incidences of gram-positive (*P* = 0.718), *Staphylococcus*-associated (*P* = 0.380), or culture negative (*P* = 0.213) peritonitis with respect to the education groups.

During follow up period, 138 (21.1%) patients developed technique failure and the average time to the technique failure was 41.2 ± 29.6 months. The causes of technique failure are shown in Table 4. Intractable peritonitis (39.9%) and mechanical problem (15.9%) were the leading causes of technique failure for all three education groups. Competing risk regression adjusted for age and gender, middle school or lower (adjusted HR, 1.87; 95% CI, 1.10–3.18; *P* = 0.038) and high school (adjusted HR, 1.95; 95% CI, 1.29–2.95; *P* = 0.002) education group were significantly associated with increased technique failure compared with the reference group (Fig 3B). In the subgroup analysis, high school group was an independent risk factor for technique failure, compared with higher than high school group in both the younger (<50 years) (adjusted HR, 1.85; 95% CI, 1.01–3.40; *P* = 0.046) and the older (≥ 50 years) (adjusted HR, 1.95; 95% CI, 1.11–3.43; *P* = 0.020) subgroups.

**Table 4:** The causes of technique failure with respect to education groups.

Cause, n (%)	All subjects (N = 655)	Education group			
		Middle school or lower (≤ 9 years) (n = 102)	High school (9<≤ 12 years) (n = 229)	Higher than high school (> 12 years) (n = 324)	*P-* value
Inadequate dialysis, n (%)	19 (13.8)	3 (10.7)	9 (14.5)	7 (14.6)	0.474
Ultrafiltration failure, n (%)	13 (9.4)	1 (3.6)	4 (6.5)	8 (16.7)	0.802
Intractable peritonitis*, n (%)	55 (39.9)	10 (35.7)	26 (41.9)	19 (39.6)	0.059
Exit-site and/or tunnel infection, n (%)	8 (5.8)	5 (17.9)	2 (3.2)	1 (2.1)	0.003
Catheter malfunction, n (%)	7 (5.1)	2 (7.1)	5 (8.14)	0 (0)	0.012
Mechanical problem, n (%)	22 (15.9)	4 (14.3)	8 (12.9)	10 (20.8)	0.917
Others, n (%)	12 (8.7)	3 (10.7)	7 (11.3)	2 (4.2)	0.040
Unknown, n (%)	2 (1.4)	0 (0)	1 (1.6)	1 (2.1)	0.100
Total	138 (100)	28 (100)	62 (100)	48 (100)	0.135

*intractable peritonitis such as refractory, recurrent, or fungal peritonitis.

## Discussion

As a home-based therapy, PD requires the self-monitoring and self-care of the patients. In the present study, middle school or lower education group was significantly associated with increased peritonitis and technique failure, compared with the reference education group. However, peritonitis was not the most important factor determining mortality in our subjects on PD. Cardiovascular events, which accounted more importantly for the PD outcome were not different between the three education groups and there was no significant difference of all-cause mortality with respect to the education levels.

A few studies showed that lower education level is significantly associated with a higher risk of first event of PD-related peritonitis, compared with higher education level [18, 19]. In our study, a similar result was observed in terms of peritonitis. The lowest education group exhibited a significantly higher risk of developing peritonitis compared with the reference group. Risk of technique failure with respect to the education levels varied among several reports. Chidambaram et al. [8] showed that lower education defined as neighborhood education level lower than high school was strong predictor of technique failure in Canadian PD patients. In contrast, Shen et al. [20] found among social factors that the education level exhibited no difference in terms of PD technique failure. Since peritonitis and exit site and/or tunnel infection combined accounted for 45.7% of total technique failure cases in our observation, the lowest education group was associated with higher risk of technique failure due to higher incidence of such infections. Therefore, it is essential to provide repeated individualized and multidisciplinary education program on self-care and self-management for PD patients. In the subgroup analysis- categorized by age < 50 or≥ 50 years—middle school or lower education group was an independent risk factor for peritonitis only in the older subgroup. No difference among the three education groups in the risk of peritonitis was observed in the younger subgroup. We speculate that in the younger subgroup, our PD training program was effective to overcome the lower academic level, while it was less effective for the older subgroup. There might be some difference in the effectiveness of training for different age groups.

One important finding we have to take notice at is that there is no significant difference in all-cause mortality among the three education groups in our study. In addition to AT analysis, *post hoc* ITT analysis was also conducted in order to preclude the possibility that the patients potentially having poorer outcomes might have been selectively censored by AT analysis. Both the AT and ITT analysis showed that lower education level was not a significant factor affecting patient survival in PD. Although lower education group in the present study showed higher risk of peritonitis during PD therapy, peritonitis accounted for only 12.8% of total deaths. Besides, overall incidence of peritonitis in our total PD patients was 0.25episodes/patient-year during follow-up period, which was remarkably lower than that reported in other studies [5, 21]. On the contrary, cardiovascular disease was the most common known cause of mortality, accounting for 33.3%, which is in keeping with other numerous reports [9, 22]. Survival of the lower education group could be improved by repeated individualized multidisciplinary education and training for the proper handling of RRT, volume and blood pressure control, and compliance with medication, *etc*.

Contrary to what might be anticipated, the incidence of *Staphylococcus*-associated peritonitis was not different but that of gram-negative peritonitis was higher in middle school or lower education group. Although gram-negative bacteria are not common as normal human skin flora of a healthy individual, gram negative bacteria may increase as skin colonizers during prolonged hospital stay or in chronically ill subjects, or patients with skin defect [23–26]. ESRD is a long-standing and chronic disease. In addition, ESRD patients could have retention of urea on the skin and experience drying of the skin due to water withdrawal [27]. Such changes may bring about a shift in skin colonizers. The authors postulated that gram-negative bacteria could increase as skin colonizers for PD patients and these results might influence on the microbiology results.

Previous studies have estimated the education level of each patient by inferring from the region of residence or area-based neighborhood education attainment, instead of obtaining the education level of the patient himself/herself [7–9]. It is the strength of our study that we included a relatively large number of only incident PD cohort–excluding prevalent patients—and obtained accurate information on the education level *per se* of each individual patient. However, there are some limitations in this study. The overall education levels of our Korean PD patients are generally higher, compared with other countries. There are no illiterate patients and very few patients had an education level lower than elementary school. Most patients have a relatively high level of education. Therefore, it is not easy to generalize the results of the present study to other countries with different socio-economic background. Education and incomes are strongly correlated with each other. Usually, patients in lower education group have lower income value, which might influence on them to get less easy access to the health care and medication. We could not obtain the direct information on the patient income. Instead, we obtained the patients’ healthcare insurance status such as medical insurance or medicaid and included these factors for adjustment. Although not perfect, such information is closely correlated with patient economic status. PD outcomes according to education level were not different after adjustment for healthcare insurance status. Lastly, since many patients (25.5%) in the present study received kidney transplant, a competing risk model treating both death and kidney transplantation as competing events would probably under-estimate the hazard of variables related to PD technique failure. However, there might be no statistical model to perfectly account for this phenomenon.

Although lower education level was significantly associated with increased peritonitis and technique failure, lower education was not associated with increased mortality in PD patients. Patients with lower education level may not be discouraged from choosing PD as their first-line RRT. Instead, overall outcomes of the lower education group could be improved by comprehensive training of PD exchange procedures and multidisciplinary education on the diet, compliance to the prescription, blood pressure control and fluid status management in patients undergoing PD therapy.

## Supporting Information

S1 File Multivariable Cox proportional hazards analysis on all-cause mortality according to education level in subgroup categorized by age < 50 (Fig A) or ≥ 50 (Fig B) years. (TIF)

S2 File Multivariable Cox proportional hazards analysis on peritonitis according to education level in subgroup categorized by age < 50 (Fig A) or ≥ 50 (Fig B) years. *Middle school or lower education group compared with higher than high school education group.
(TIF)
