# Supplementary figures and images for: Lower Education Level Is a Risk Factor for Peritonitis and Technique Failure but Not a Risk for Overall Mortality in Peritoneal Dialysis under Comprehensive Training System
Source: PLoS One. 2017 Jan 5;12(1):e0169063. doi: 10.1371/journal.pone.0169063 (PMC5215932; doi:10.1371/journal.pone.0169063)

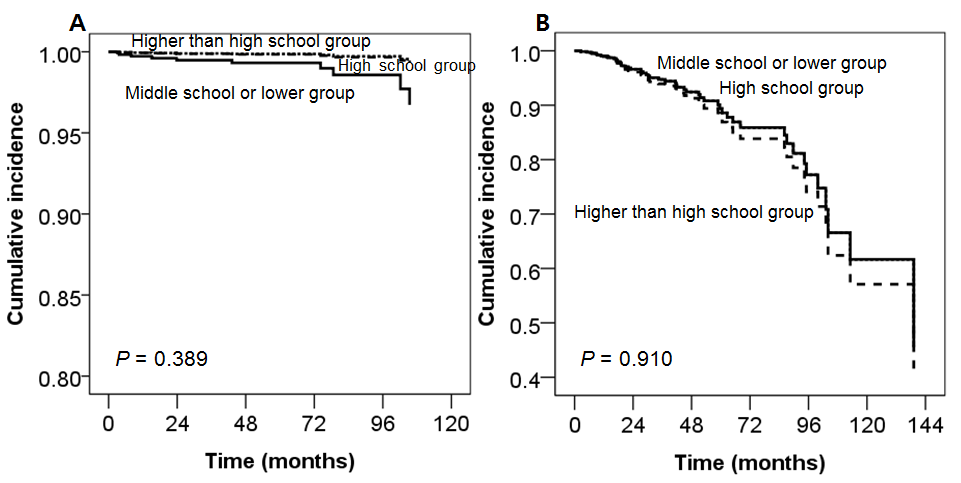

Supplement: S1 File — (TIF) [file pone.0169063.s001.tif]

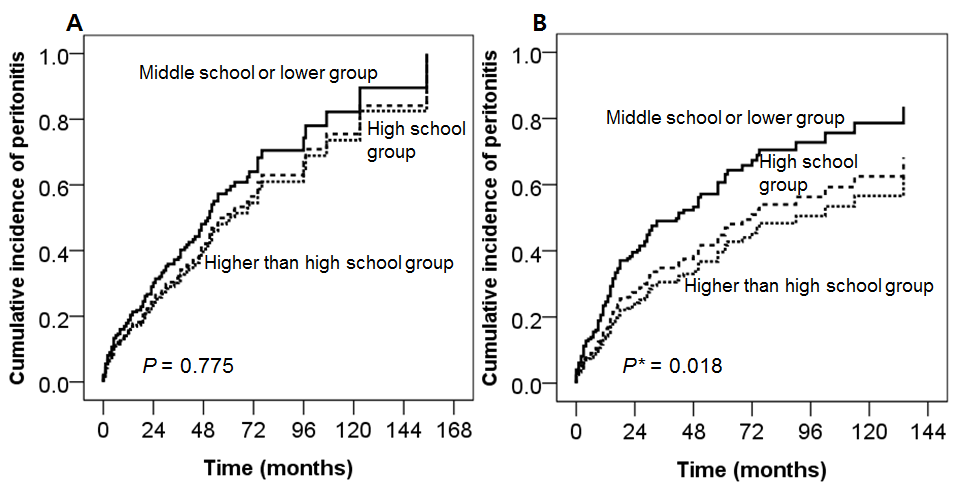

Supplement: S2 File — *Middle school or lower education group compared with higher than high school education group. (TIF) [file pone.0169063.s002.tif]
